# Foot and Mouth Disease and Cryptosporidiosis: Possible Interaction between Two Emerging Infectious Diseases

**DOI:** 10.3201/eid0901.020265

**Published:** 2003-01

**Authors:** Paul R. Hunter, Rachel M. Chalmers, Qutub Syed, L. Sara Hughes, Sarah Woodhouse, Louise Swift

**Affiliations:** * University of East Anglia, Norwich, United Kingdom; † Singleton Hospital, Swansea, United Kingdom; ‡ Communicable Disease Surveillance Centre, Chester, United Kingdom

**Keywords:** cryptosporidiosis, *Cryptosporidium parvum*, foot and mouth disease, incidence, zoonosis, dispatch

## Abstract

During 2001, a large outbreak of foot and mouth disease occurred in the United Kingdom, during which approximately 2,030 confirmed cases of the disease were reported, >6 million animals were slaughtered, and strict restrictions on access to the countryside were imposed. We report a dramatic decline in the reported incidence of human cryptosporidiosis in northwest England during weeks 13–38 in 2001, compared with the previous 11 years. This decline coincided with the period of foot and mouth restrictions. No similar reduction occurred in the other 26 weeks of the year. We also noted a substantial decline in the proportion of human infections caused by the bovine strain (genotype 2) of *Cryptosporidium parvum* during weeks 13–38 that year but not during the other weeks.

Cryptosporidiosis is an acute diarrheal disease caused by a protozoan parasite *Cryptosporidium parvum* (*1*). Although the disease is self-limiting in most instances, in certain immunocompromised patients the infection can be very severe and potentially fatal (*2*). This disease is now the most common parasitic cause of human diarrheal disease in the United Kingdom; over the last 10 years, the northwest region of England has regularly reported more cases than any other region in the country (*3*). Cryptosporidiosis was originally thought to be a zoonosis, but epidemiologic studies (*4*) and the description of two *C. parvum* genotypes, one of which was only found in humans (genotype 1 or H), has highlighted the importance of person-to-person transmission (*5*). Nevertheless, zoonotic transmission is still an important route of infection, though the proportion of human *Cryptosporidium* infections originating from animals is still unknown.

During 2001, a major outbreak of foot and mouth disease occurred in the United Kingdom. Coincident with this outbreak, we noted a dramatic decline in reports of *Cryptosporidium* infection in the northwest region. We describe the change in epidemiology of reported cryptosporidiosis during that year and discuss the hypothesis that this reduction may have resulted from public health measures introduced to control the foot and mouth disease epidemic.

## Foot and Mouth Disease Epidemic in 2001

**Figure 1:**
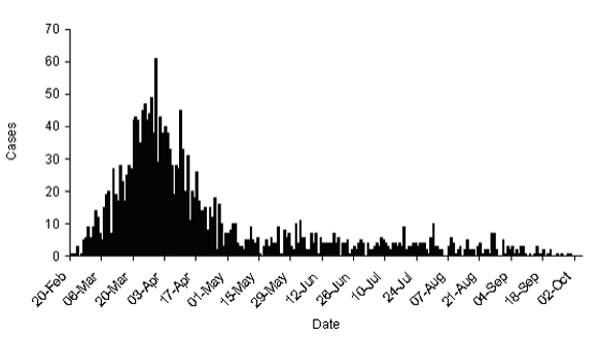
Epidemic curve of the foot-and-mouth disease epidemic, United Kingdom, 2001.

During 2001, the United Kingdom experienced its largest recorded outbreak of foot and mouth disease. The first case was identified on February 19 in sows awaiting slaughter at an abattoir in Essex County, in the south of England. The epidemic reached its peak on March 30 when 61 new cases were identified. Although most cases had occurred by the end of April, the final case was not identified until September 30, for a total of 2,030 confirmed cases (Figure 1) (*6*). A case was defined as infection in one or more animals on a single premises.

To control the epidemic, **4,196,580** animals were slaughtered (*7*). Animals were slaughtered if they were on infected premises, on farms neighboring infected premises, on premises with animals that had direct contact with infected animals, and if any infection was suspected. In addition, 2,048,769 animals were slaughtered under the livestock welfare (disposal) scheme as movement of animals was otherwise banned. In total, 6,245,349 animals were slaughtered. Officials disposed of approximately 600,000 tons of carcasses: approximately 130,000 by rendering; 95,000 in licensed commercial landfill sites; and 61,000 at four mass burial sites (*8*). Burial of carcasses occurred on >900 farms and burning on >950 farms. Approximately 100,000 tons of pyre ash were transferred to landfills. Specific details on the timing of slaughter are not available, although because most slaughtering occurred in response to, and soon after, the diagnosis of new cases, the slaughter curve would be expected to follow the epidemic curve (Figure 1).

In addition, widespread bans were set on the movement of animals and human access to the countryside was restricted. The first restriction order in the United Kingdom was issued on February 21, covering parts of Essex and Kent in southern England. The first restriction order in the northwest region was issued 6 days later. Thereafter, new orders were issued as new cases were identified. Initially, most counties in or near infected areas imposed strict and widespread bans on access to the countryside, although the central government subsequently persuaded most counties to relax their most rigorous bans except in areas of confirmed disease activity. Beginning in early June, public rights of way started to be reopened; by July 27, an estimated 85% of public rights of way were open again. However, even after restrictions were lifted, fewer persons visited the countryside during the summer of 2001. More details, including maps of the distribution of cases, can be obtained from the Department of the Environment, Food and Rural Affairs (available from: URL: www.defra.gov.uk/footandmouth).

In general, restrictions were lifted when local areas had been free of infection for 6–8 weeks. The final case of foot and mouth disease diagnosed, on September 30, was in the northwest region of England. The last restrictions were lifted on November 19.

## Human Cryptosporidiosis

Data used in this study came from routine reports to the Communicable Disease Surveillance Centre–North West (CDSC–NW) and from isolate submissions to the Public Health Laboratory Service (PHLS) Cryptosporidium Reference Unit. These reports and isolates come from both National Health Service and PHLS microbiology laboratories in the region. Case-patients were defined as persons who visited a physician’s office or hospital because of diarrhea and provided a stool sample that tested positive for *Cryptosporidium*. All laboratories in the northwest region report their positive stool sample results to CDSC–NW, usually electronically.

The area covered by the northwest region is home to 6.6 million people, 65% of whom live in the large urban areas of Liverpool and Manchester. The central and southern parts of the region have both cattle and sheep farming. The northern part of the region covers the southern English Lake District in the county of Cumbria, where the main industries are tourism and sheep farming.

**Figure 2:**
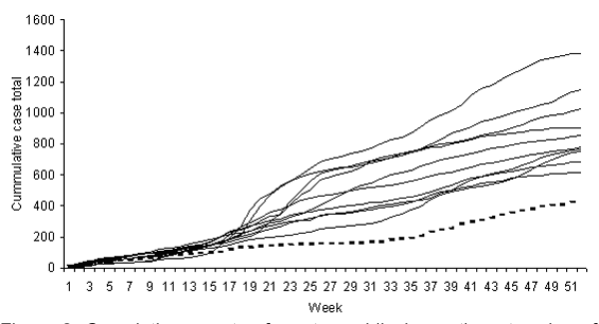
Cumulative reports of cryptosporidiosis, northwest region of England, 1990–2001. Broken line indicates data for 2001; other lines indicate data for 1990–2000.

Figure 2, which shows the number of reports for 1991–2001, depicts cumulative totals by week for each year, providing the total number of cases in each year up to and including the week indicated. The key observation is the virtually flat slope of the curve for weeks 17 to 32, indicating that very few cases occurred during that time.

Defining the period when foot and mouth disease controls were in place across the region was difficult because controls were imposed at different times at each locality, reflecting the progress of the epidemic. The index case was identified in the last week of February (week 9), but controls were not widespread until a few weeks later. We chose to designate week 13 (week beginning March 26, 2001) as the first week in which any controls would likely have an impact on laboratory reports of cryptosporidiosis. The incubation period for cryptosporidiosis is long (1–2 weeks), and often delays occur in submitting samples to the laboratory and the subsequent reporting of cases by the laboratories. From our past experience of outbreak investigations, we know that the time from the causative event to detectable changes in laboratory reporting is 3–4 weeks (*9*). We then arbitrarily deemed the 26 weeks after week 13 to be the period when controls were in place. Reported cases for weeks 13–38 in 2001 were then compared with the same weekly period for the previous 11 years. We made this same comparison for the remaining 26 weeks of each year (weeks 1–12 and 39–52).

Reports during weeks 13–38 were substantially lower than in previous years, though not for the other weeks in the year (Figure 2) (Table 1). To determine the strength of this reduction, we calculated a t-test value as follows: 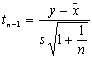, where *y* is the value for 2001, *n*=number, *s=*standard deviation, and 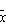
=mean of the previous 10 years. The number of reports during weeks 13–38 was significantly lower in 2001 compared with reports during those weeks in the previous 10 years (*t*_9_= –1.993, p=0.039), but the number of reports for the other weeks of 2001 was not significantly lower (*t*_9_ = –0.384, p=0.355).

**Table 1:** Total reported cases of human cryptosporidiosis in time periods, northwest England, 1990–2001

Yr	Total cases for England and Wales	Cases reported in northwest		
		Total for yr	Wks 13–38	Other weeks in yr
1991	5,165	768	372	396
1992	5,211	1,151	708	443
1993	4,832	850	577	273
1994	4,432	683	387	296
1995	5,691	750	367	383
1996	3,660	612	353	259
1997	4,321	1,023	701	322
1998	3,745	777	517	260
1999	4,759	903	722	181
2000	5,799	1,382	872	510
Mean for 1991–2000	4,762	890	558	332
2001	3,681	428	159	269

During 2001, 428 cryptosporidiosis cases were reported during the year compared with 1,382 cases in 2000 (69.0% decline). For the period from weeks 13–38, 159 cases were reported compared with 872 reports in 2000 (81.8% decline). Throughout England and Wales, a 36.5% reduction in cases for 2001 was reported compared with 2000 (from 5,799 to 3,681) (*10*).

Strains of *Cryptosporidium* from those infected in the northwest region are generally sent to the Cryptosporidium Reference Laboratory at Swansea Public Health laboratory for typing. However, laboratories use varied criteria to decide which strains to send for typing, and not all laboratories send strains. Strains were genotyped by using polymerase chain reaction and restriction fragment length polymorphism analysis of a region of the *Cryptosporidium* oocyst wall protein gene (*11*). The results of typing for 2000 and 2001, the only years with typing data available, are shown in Table 2. A significant decline in the proportion of strains due to the bovine genotype (compared to all others) occurred in weeks 13–38 in 2001 compared with weeks 13–38 in 2000 (chi square=20.01, p=0.000008) but did not occur during the other 26 weeks (chi square=3.68, p=0.06).

**Table 2:** Distribution of genotypes of *Cryptosporidium parvum*, United Kingdom, 2000 and 2001

Wks	Yr	*C. parvum* genotype			Total
		1 (% human)	2 (% bovine)	Other (%)	
13–38	2000	185 (29)	440 (69)	9 (2)	634
	2001	59 (42)	70 (50)	12 (8)	141
1–12 and 39–52	2000	171 (62)	96 (35)	10 (4)	277
	2001	148 (70)	56 (27)	7 (3)	211

## Conclusions

Most of what is known about the epidemiology of *Cryptosporidium* infections comes from outbreak investigations that have generally highlighted drinking water and recreational contact with water as major sources (*12*). Despite the considerable interest in *Cryptosporidium* in both the United States and the United Kingdom in recent years, very little is known about the epidemiology of sporadic infection with this organism.

The conclusion that the decline in cases was related to the outbreak of foot and mouth disease is warranted, as the decrease in expected reports was almost coincident with the introduction of control measures (after an appropriate lag to account for incubation period and reporting delay). The mechanism for this decline is unclear. The decline is likely to be real and not due to reduced efficiency of the surveillance system because reports of infection with *Campylobacter*, the most commonly reported enteric pathogen, showed no similar reduction in 2001 compared with reports in 2000.

Following several outbreaks of disease linked to a single water supply system (*13*), the local water utility has implemented a number of control measures; these measures likely also had an effect. However, the control measures imposed on a single water supply could not explain the decline seen throughout the region or elsewhere in the United Kingdom.

Another explanation may be the decline in animal population as a result of the slaughter policy. However, marked reductions in cases were seen, even in those areas where there were relatively few animals slaughtered. For example, in the three northern health-authority areas where most cases occurred, 299 cases of cryptosporidiosis occurred during weeks 13–38 in 2000 whereas in 2001, 44 cases occurred (an 85% reduction). In Cheshire, where relatively few cases were identified, the respective figures were 35%, 12%, and 66%. In our view, the most likely explanation for the decline in cases of cryptosporidiosis was the removal of access to the countryside, which prevented humans from coming into contact with farm and wild animals and their excrement.

The surveillance data presented support previous evidence that zoonotic transmission is a major route of infection in this region (*14*). However, caution must be used when extrapolating this experience to the rest of the United Kingdom. The fact that one third of cryptosporidia detected in England are of human only (genotype 1) type has been described (*14*). Also, the relative distribution of genotypes 1 and 2 in England varies from region to region with the northwest region having the highest proportion of type 2 (bovine) strains detected. Consequently, the high proportion of infections being suggested as zoonotic in this report would not necessarily apply elsewhere.
